# Serum matrix metalloproteinase-7 as a diagnostic and prognostic biomarker in primary biliary cholangitis

**DOI:** 10.3389/fmed.2026.1821563

**Published:** 2026-04-22

**Authors:** Jiahui Chen, Xiaoying Shang, Chong Peng, Luan Wang, Nana Zhang, Wenlong Lu, Mingjun Liu

**Affiliations:** 1 Department of Clinical Laboratory, The Affiliated Hospital of Qingdao University, Qingdao, Shandong, China; 2 Department of Clinical Laboratory, Zibo Hospital of Traditional Chinese Medicine, Zibo, Shandong, China; 3 Department of Clinical Laboratory, Qingdao Municipal Hospital, Qingdao, Shandong, China

**Keywords:** biomarker, cirrhosis, liver fibrosis, matrix metalloproteinase-7, primary biliary cholangitis

## Abstract

**Objectives:**

This study aimed to assess the diagnostic and prognostic value of serum matrix metalloproteinase-7 (MMP-7) in patients with primary biliary cholangitis (PBC).

**Methods:**

Serum MMP-7 was measured using chemiluminescence immunoassay in 184 PBC patients and 94 healthy controls. PBC patients were stratified into cirrhotic (*n* = 75) and non-cirrhotic (*n* = 109) subgroups; 65 underwent liver biopsy with Ludwig classification. Liver function tests, platelet count, the aspartate aminotransferase-to-platelet ratio index (APRI), and fibrosis-4 index (FIB-4) were assessed. Diagnostic performance was evaluated via group comparisons, Spearman correlation, and receiver operating characteristic curve analysis.

**Results:**

MMP-7 levels were significantly higher in PBC patients compared with healthy controls [5.12 (3.73–6.97) vs. 2.46 (1.93–2.84) ng/mL, *p* < 0.001], and further elevated in cirrhotic vs. non-cirrhotic [6.99 (5.80–11.80) vs. 4.11 (3.59–5.25) ng/mL] and in advanced vs. early fibrosis [7.13 (5.78–11.02) vs. 3.83 (3.52–5.61) ng/mL; *p* < 0.001]. For cirrhosis discrimination, MMP-7 achieved an area under the curve (AUC) of 0.830 (95% CI: 0.765–0.894); combining it with APRI/ FIB-4 did not significantly improve accuracy (combined AUC = 0.849, *p* = 0.224). For advanced fibrosis, AUC was 0.877 (95% CI: 0.794–0.959). MMP-7 correlated with APRI, FIB-4, and histological stage (*p* < 0.01). After 3–4 months of ursodeoxycholic acid treatment, MMP-7 decreased significantly and paralleled alkaline phosphatase decline.

**Conclusion:**

Serum MMP-7 is elevated in PBC and correlates with disease severity, including cirrhosis and advanced fibrosis. It shows robust diagnostic performance as a standalone biomarker and demonstrates promise for monitoring treatment response, underscoring its potential clinical utility as a complementary non-invasive tool in PBC management.

## 1 Introduction

Primary biliary cholangitis (PBC) is an autoimmune liver disorder characterized by the progressive destruction of intrahepatic bile ducts and cholestasis, which can ultimately lead to liver fibrosis, cirrhosis, and hepatic failure (1). The disease typically follows an insidious course, with patients often remaining asymptomatic until advanced stages (2). Notably, even when liver fibrosis or early cirrhosis is established, timely and standardized treatment may still halt, attenuate, or even reverse its progression (3, 4). Consequently, the accurate assessment of disease stage is paramount for effective management and improving long-term patient outcomes.

Currently, liver biopsy remains the histological gold standard for staging liver fibrosis and assessing disease activity. However, its inherent invasiveness, risk of sampling error, and inability to facilitate dynamic monitoring limit its routine application, confining its use primarily to cases with diagnostic uncertainty or a need for precise staging (1, 5). Non-invasive tools, such as the aspartate aminotransferase-to-platelet ratio index (APRI) and the fibrosis-4 index (FIB-4), have been widely adopted for fibrosis assessment (6, 7). Nevertheless, their diagnostic accuracy in PBC can be compromised by various confounders (e.g., concurrent hematological disorders, medication use) and the lack of well-validated, disease-specific cut-off values (7). Moreover, although ursodeoxycholic acid (UDCA) remains the first-line therapy for PBC, a substantial proportion of patients exhibit suboptimal biochemical response, and treatment options for advanced disease remain limited (8, 9). This underscores the urgent need for reliable non-invasive biomarkers specifically validated in PBC to facilitate early identification of high-risk patients and enable timely therapeutic intervention.

Matrix metalloproteinases are zinc-dependent endopeptidases that require calcium for their activity. They play a crucial role in the degradation of the hepatic extracellular matrix, directly or indirectly contributing to both the development and resolution of liver fibrosis (10). Serum matrix metalloproteinase-7 (MMP-7), an important member of the matrix metalloproteinases family, is produced by various cell types including cholangiocytes, hepatic stellate cells, and macrophages (Kupffer cells) (11–13). MMP-7 exerts a central role in tissue remodeling and fibrogenesis by degrading multiple extracellular matrix components, such as type IV collagen and fibronectin (14), and by modulating key profibrotic signaling pathways, including TGF-*β* (15–17). Notably, MMP-7 can be specifically upregulated in injured biliary epithelial cells, suggesting its direct involvement in the pathological progression from bile duct damage to liver fibrosis (18), which makes it a promising biomarker for PBC. Although the diagnostic value of MMP-7 has been established in biliary atresia (19, 20), its behavior in PBC—a disease characterized by autoimmune cholangitis as the core pathological feature—remains poorly understood. Specifically, the relationship between serum MMP-7 levels and histological fibrosis stages or clinical cirrhosis, its diagnostic performance for advanced fibrosis compared with existing non-invasive models, and its dynamic changes in response to UDCA treatment have not been comprehensively evaluated in clinical cohorts.

Therefore, this study aimed to systematically evaluate the clinical significance of serum MMP-7 in PBC. Specifically, we sought to: (1) determine its circulating levels and assess their association with disease severity, including clinically defined cirrhosis and histologically staged fibrosis; (2) compare its diagnostic accuracy for advanced disease against established non-invasive scores (APRI and FIB-4); and (3) preliminarily explore its dynamics in response to UDCA treatment. By addressing these aspects, our work provides a comprehensive assessment of MMP-7 not only as a diagnostic biomarker but also as a potential tool for monitoring disease progression and treatment response in PBC, filling a notable gap in current non-invasive diagnostic strategies.

## 2 Materials and methods

### 2.1 Study design and participants

This single-center retrospective cohort study was conducted at the Affiliated Hospital of Qingdao University. A total of 184 patients diagnosed with PBC between December 2022 and October 2025 were enrolled as the disease group. Based on clinical and imaging assessments, these patients were further stratified into a cirrhosis subgroup (*n* = 75) and a non-cirrhosis subgroup (*n* = 109). A total of 94 healthy individuals who underwent routine health examinations during the same period served as the healthy control group.

### 2.2 Diagnostic criteria for PBC

The diagnosis of PBC was established according to the 2018 American Association for the Study of Liver Diseases practice guidelines (1), which require at least two of the following three criteria:

1. Biochemical evidence of cholestasis, primarily based on elevated serum alkaline phosphatase (ALP) levels;
2. Presence of serum anti-mitochondrial antibodies (AMA); or, in AMA-negative cases, the presence of other PBC-specific autoantibodies (e.g., anti-sp100 or anti-gp210);
3. Histopathological findings on liver biopsy consistent with non-supportive destructive cholangitis and interlobular bile duct destruction.

### 2.3 Inclusion and exclusion criteria

All participants in the PBC group met the diagnostic criteria outlined above. To specifically assess treatment response, a subgroup for treatment response analysis (*n* = 33) was defined. This subgroup comprised patients from the main cohort who had a confirmed PBC diagnosis and received continuous, standard-dose ursodeoxycholic acid (UDCA) therapy for approximately 3 months from the baseline assessment. Within this subgroup, 27 patients had two serial measurements (baseline and post-treatment), and 6 patients underwent multiple consecutive measurements.

Individuals meeting any of the following criteria were excluded from the study:

1. Co-infection with other hepatotropic viruses (e.g., hepatitis B virus or hepatitis C virus);
2. A history of alcoholic liver disease or metabolic dysfunction-associated steatotic liver disease;
3. Diagnosis of autoimmune hepatitis–PBC overlap syndrome or other systemic autoimmune disorders;
4. Severe renal, pulmonary, or other major organ dysfunction;
5. Active malignancy or any other severe systemic condition deemed likely to significantly confound serum biomarker levels.

### 2.4 Clinical diagnosis of cirrhosis

Patients were classified into the cirrhosis group if they met any of the following criteria:

1. Histological confirmation of cirrhosis on liver biopsy.
2. Presence of typical imaging features of cirrhosis on abdominal ultrasound, computed tomography, or magnetic resonance imaging (e.g., nodular liver surface or lobular disproportion) concurrent with signs of portal hypertension (e.g., splenomegaly, portal vein diameter >13 mm, or the presence of esophageal/gastric varices).

### 2.5 Histological staging

Liver biopsy was performed in 65 PBC patients based on standard clinical indications. All biopsy specimens were independently assessed by two experienced hepatopathologists who were blinded to all clinical and biochemical data, using the Ludwig histological staging system. According to this system, fibrosis was staged from I to IV. For analysis, patients were categorized into two groups: early fibrosis (Ludwig stages I-II, indicating portal inflammation/periportal fibrosis; *n* = 27) and advanced fibrosis (Ludwig stages III-IV, indicating bridging fibrosis or cirrhosis; *n* = 38). This stratification was performed to validate serum MMP-7 levels against the histological gold standard.

### 2.6 Sample collection and storage

Residual serum samples from routine clinical tests were collected and aliquoted. Aliquots of 300 μL were stored at −80 °C and subjected to a single freeze–thaw cycle to preserve stability.

### 2.7 Measurement of serum MMP-7

Serum MMP-7 concentrations were measured using a chemiluminescence immunoassay kit (Shenzhen New Industries Biomedical Engineering Co., Ltd., Shenzhen, China; Catalog number: [100825031101]). This kit is intended for research use only and has not yet been approved for clinical diagnostic use by the National Medical Products Administration of China. All procedures were performed in strict accordance with the manufacturer’s protocol.

### 2.8 Measurement of routine laboratory parameters

Standard liver function tests, including aspartate aminotransferase (AST), alanine aminotransferase (ALT), gamma-glutamyl transferase (GGT), alkaline phosphatase (ALP), albumin (ALB), total bilirubin (TBIL), and direct bilirubin (DBIL), were performed on a Hitachi 7,600 automatic biochemical analyzer using corresponding WAKO diagnostic reagents. Platelet count (PLT) was determined using a Sysmex automated hematology analyzer with its proprietary reagents.

### 2.9 Calculation of non-invasive fibrosis scores

APRI and FIB-4 were calculated using the following equation (21):


$$\begin{array}{l} \text{APRI}=(\mathrm{AST}/\mathrm{ULN})\times 100/\mathrm{PLT};\mathrm{FIB}-4\\ =\mathrm{age}\times \mathrm{AST}/(\mathrm{PLT}\times \sqrt{\mathrm{ALT}}). \end{array}$$

### 2.10 Statistical analysis

Statistical analyses were performed using Statistical Package for the Social Sciences (version 27.0; IBM Corp., Armonk, NY, USA) and GraphPad Prism (version 10; GraphPad Software, San Diego, CA, USA). Continuous variables with a normal distribution are presented as mean ± standard deviation and were compared using the independent Student’s t-test or one-way analysis of variance. Non-normally distributed data are presented as median with interquartile range (IQR) and were compared using the Mann–Whitney U test or the Kruskal-Wallis H test. The Wilcoxon signed-rank test was used for paired comparisons before and after treatment. Correlation analyses were conducted using Pearson’s test for normally distributed variables and Spearman’s rank test for non-normally distributed variables. Categorical data are presented as numbers and percentages, n (%). Between-group comparisons were performed using the Pearson chi-square (*χ*^2^) test. We evaluated the diagnostic efficacy of individual biomarkers and their combinations using binary logistic regression analysis, with performance assessed by receiver operating characteristic (ROC) curves. As this was an exploratory study intended for hypothesis generation rather than confirmatory validation, no post-hoc pairwise subgroup comparisons were conducted, and no adjustments for multiple testing were applied for any analyses. A two-tailed *p* value < 0.05 was considered statistically significant.

## 3 Results

### 3.1 Baseline characteristics and serum MMP-7 levels across PBC disease stages

The study comprised 184 patients with PBC and 94 healthy controls. Baseline characteristics are detailed in Table 1. The cohort exhibited the expected biochemical profile of PBC, with significant elevations in cholestatic markers (ALP, GGT, TBIL, DBIL) and reductions in albumin and platelets compared to healthy individuals (*p* < 0.001).

**Table 1:** Clinical and laboratory characteristics of the study population.

Variant	Healthy controls (*n* = 94)	PBC (All) (*n* = 184)	PBC (Non-cirrhotic) (*n* = 109)	PBC (Cirrhotic) (*n* = 75)	*p* value (HC vs. PBC-all)	*p* value (PBC-non-cirrhotic vs. PBC-cirrhotic)
Age (y)	55.87 ± 11.17	57.20 ± 10.67	54.35 ± 9.53	61.35 ± 10.92	0.334	<0.0001
Sex					0.666	0.194
Male	44 (47.62%)	13 (7.07%)	6 (5.50%)	8 (10.67%)		
Female	50 (52.38%)	171 (92.93%)	103 (94.50%)	67 (89.33%)		
AST (U/L)	19.00 (15.75–22.00)	25.00 (20.00–36.00)	23.00 (19.50–29.70)	30.00 (24.00–46.00)	<0.0001	<0.0001
ALT(U/L)	14.50 (11.00–19.00)	24.00 (16.00–34.75)	22.00 (14.50–29.50)	28.00 (19.00–39.00)	<0.0001	0.005
GGT (U/L)	15.00 (11.00–19.00)	36.00 (19.00–80.50)	30.00 (18.00–59.50)	47.00 (24.00–114.00)	<0.0001	0.002
ALP (U/L)	58.46 ± 11.23	104.00 (76.25–137.75)	95.00 (69.50–122.50)	119.00 (90.00–169.00)	<0.0001	<0.0001
ALB (g/L)	46.52 ± 2.28	43.70 (39.63–45.60)	44.60 (42.40–46.40)	40.50 (35.60–44.30)	<0.0001	<0.0001
TBIL (μmol/L)	10.60 (7.63–13.79)	14.08 (10.78–21.38)	12.25 (10.36–15.88)	19.91 (13.40–25.07)	<0.0001	<0.0001
DBIL (μmol/L)	3.64 (2.80–4.42)	5.17 (3.96–8.18)	4.59 (3.68–6.12)	7.89 (4.71–12.13)	<0.0001	<0.0001
PLT (×10^9^/L)	253.45 ± 51.36	195.00 (134.75–246.75)	214 (166.00–258.50)	150.00 (101.00–200.00)	<0.0001	<0.0001
APRI	0.21 (0.17–0.26)	0.38 (0.28–0.83)	0.34 (0.25–0.49)	0.75 (0.37–1.37)	<0.0001	<0.0001
FIB-4	1.03 (0.92–1.37)	1.63 (1.08–2.88)	1.31 (0.97–2.02)	2.89 (1.55–5.41)	<0.0001	<0.0001
MMP-7 (ng/mL)	2.46 (1.93–2.84)	5.12 (3.73–6.97)	4.11 (3.59–5.25)	6.99 (5.80–11.80)	<0.0001	<0.0001

Data are presented as mean ± standard deviation, median (IQR), or *n* (%). PBC, primary biliary cholangitis; AST, aspartate aminotransferase; ALT, alanine aminotransferase; GGT, gamma-glutamyltransferase; ALP, alkaline phosphatase; ALB, albumin; TBIL, total bilirubin; DBIL, direct bilirubin; PLT, platelet; APRI, aspartate aminotransferase-to-platelet ratio index; FIB-4, fibrosis-4 index; MMP-7, matrix metalloproteinase-7.

**Figure 1:**
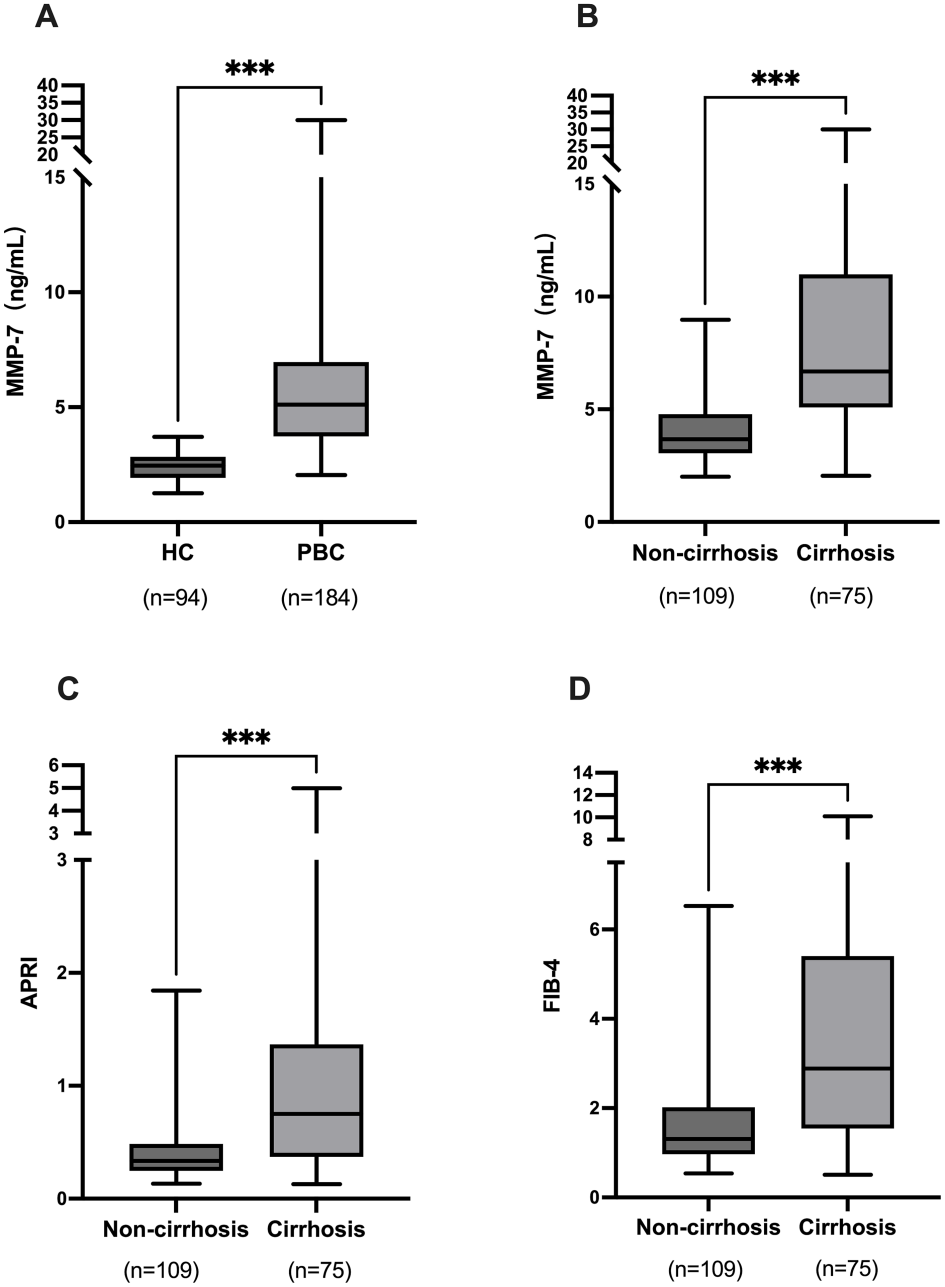
Comparison of serum MMP-7 levels and conventional fibrosis scores across PBC disease stages. **(A)** MMP-7 concentrations in healthy controls (HC) versus patients with PBC. **(B)** MMP-7 in non-cirrhotic versus cirrhotic PBC patients. **(C)** APRI scores in non-cirrhotic versus cirrhotic PBC patients. **(D)** FIB-4 scores in non-cirrhotic versus cirrhotic PBC patients. ***p < 0.001. MMP-7, matrix metalloproteinase-7; APRI, aspartate aminotransferase-to-platelet ratio index; FIB-4, fibrosis-4 index.

Critically, serum MMP-7 concentrations were more than two-fold higher in PBC patients than in controls [median: 5.12 (IQR: 3.73–6.97) vs. 2.46 (1.93–2.84) ng/mL, *p* < 0.001] (Figure 1A). When patients were stratified by cirrhosis status, MMP-7 demonstrated a striking stepwise increase, with cirrhotic patients showing concentrations approximately 70% higher than their non-cirrhotic counterparts [6.99 (5.80–11.80) vs. 4.11 (3.59–5.25) ng/mL, *p* < 0.001] (Figure 1B). This gradient was also observed—though less markedly—with conventional fibrosis scores APRI and FIB-4 (Figures 1C,D), establishing a clear severity continuum for biomarker evaluation.

### 3.2 Analysis of serum MMP-7 levels based on Ludwig pathological staging

Histological evaluation was performed on the 65 PBC patients who underwent liver biopsy. According to the Ludwig classification system, the patients were distributed as follows: Stage I (*n* = 9), Stage II (*n* = 18), Stage III (*n* = 11), and Stage IV (*n* = 27). The Kruskal-Wallis test revealed a statistically significant overall difference in serum MMP-7 levels across these pathological stages (*H* = 31.334, *p* < 0.001).

**Figure 2:**
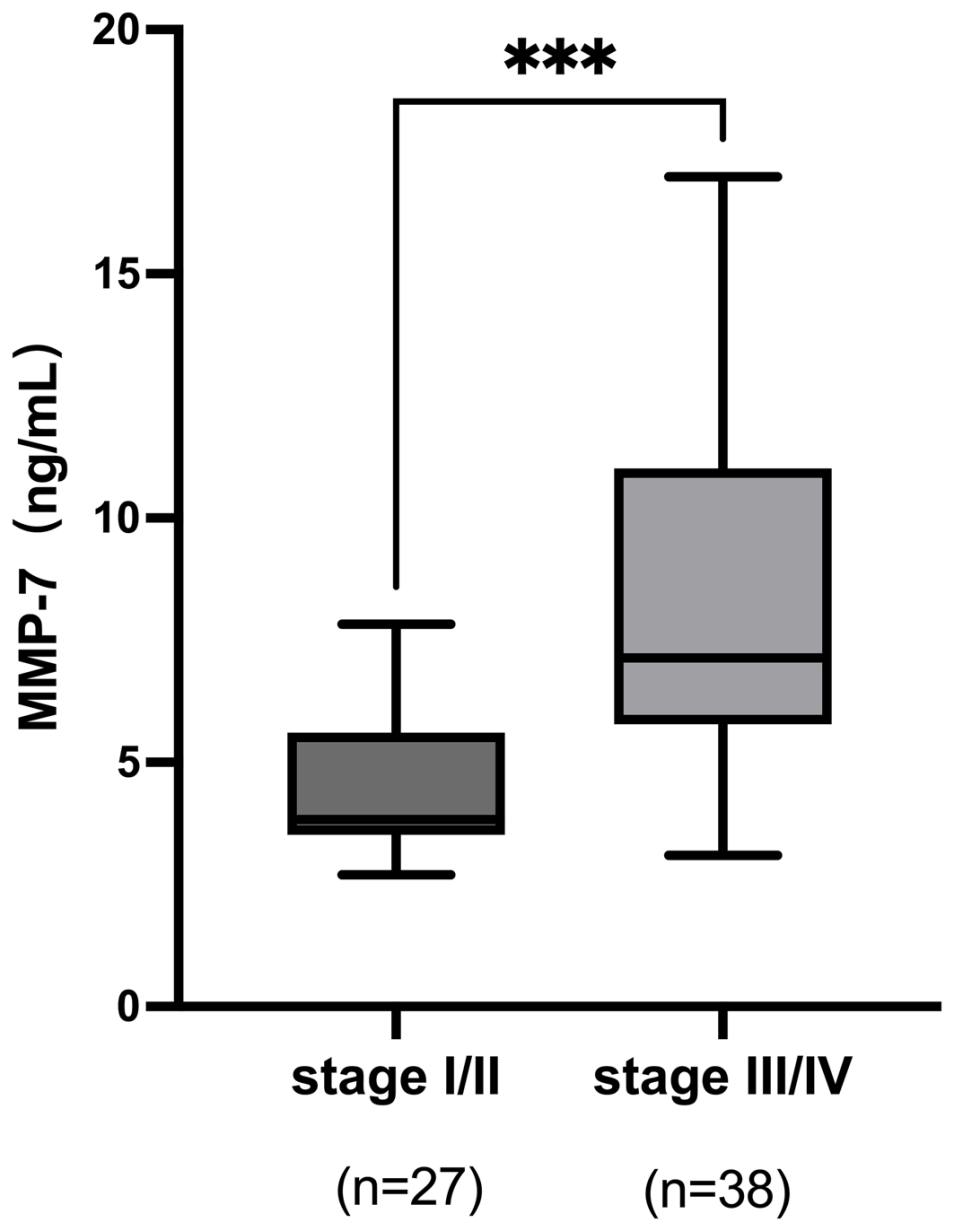
Distribution of serum MMP-7 levels stratified by Ludwig histological stage. ***p < 0.001.

For analytical purposes and to ensure adequate statistical power in group comparisons, patients were consolidated into two cohorts: early fibrosis (Ludwig stages I-II, *n* = 27) and advanced fibrosis (Ludwig stages III-IV, *n* = 38). The Mann–Whitney *U* test demonstrated that the median serum MMP-7 concentration was significantly higher in the advanced fibrosis cohort [7.13 (5.78–11.02) ng/mL] than in the early fibrosis cohort [3.83 (3.52–5.61) ng/mL] (*p* < 0.001; Figure 2).

### 3.3 Diagnostic performance of serum MMP-7 for advanced disease in PBC

Diagnostic stratification followed two distinct pathways. Clinically, 75 patients met criteria for cirrhosis based on predefined clinical and imaging assessments, while 109 were classified as non-cirrhotic. Histologically, the 65 patients with available liver biopsies were staged using the Ludwig classification and grouped into early fibrosis (stages I-II, *n* = 27) and advanced fibrosis (stages III-IV, *n* = 38). With these well-defined cohorts, serum MMP-7 demonstrated robust diagnostic accuracy across both clinical and histological endpoints.

**Figure 3:**
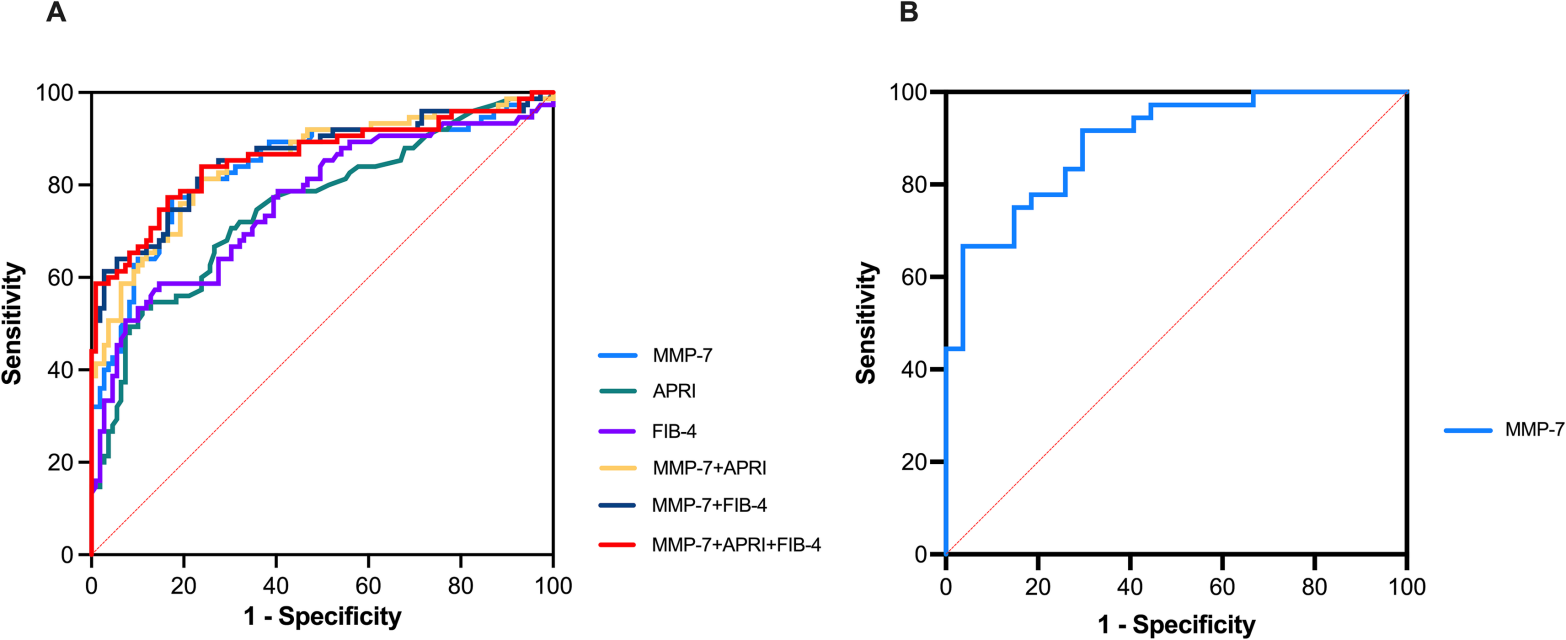
ROC curves of MMP-7 and other indices for diagnosing advanced PBC. **(A)** ROC curves for differentiating cirrhotic PBC from non-cirrhotic PBC using MMP-7 and non-invasive fibrosis models. **(B)** ROC curve analysis of serum MMP-7 for discriminating advanced liver fibrosis in patients with PBC. ROC, receiver operating characteristic; MMP-7, matrix metalloproteinase-7; APRI, aspartate aminotransferase-to-platelet ratio index; FIB-4, fibrosis-4 index.

For discrimination of cirrhosis, MMP-7 achieved an area under the curve (AUC) of 0.830 (95% CI: 0.765–0.894). The optimal cutoff, determined by the Youden index, was 5.79 ng/mL, yielding a sensitivity of 76.0% and a specificity of 81.7%. MMP-7 significantly outperformed APRI (AUC = 0.749, *p* = 0.045) and showed a trend toward superiority over FIB-4 (AUC = 0.759, *p* = 0.080) (Figure 3A).

For detection of advanced histological fibrosis (Ludwig stages III-IV), MMP-7 demonstrated even higher diagnostic accuracy, with an AUC of 0.877 (95% CI: 0.794–0.959). At the Youden index derived cutoff of 6.48 ng/mL, specificity reached 96.3% with a sensitivity of 65.8% (Figure 3B).

Notably, combining MMP-7 with APRI or FIB-4 did not significantly improve diagnostic performance over MMP-7 alone (combined AUC = 0.849, *p* = 0.224), underscoring its standalone utility. Detailed metrics are provided in Table 2.

**Table 2:** Diagnostic performance of MMP-7 and non-invasive models in differentiating cirrhosis from non-cirrhosis in PBC.

Variable	Cut-off	AUC	95%CI	Youden index	SE%	SP%	*p* (vs. MMP-7)
MMP-7 (ng/mL)	5.79	0.830	0.765–0.894	0.577	76.0	81.7	1.000
APRI	0.72	0.749	0.675–0.822	0.401	52	88.1	0.045
FIB-4	2.41	0.759	0.686–0.833	0.445	57.3	87.2	0.080
MMP-7 + APRI	0.45	0.838	0.777–0.899	0.542	68.0	86.2	0.454
MMP-7 + FIB-4	0.64	0.849	0.788–0.911	0.585	61.3	97.2	0.224
MMP-7 + APRI+FIB-4	0.38	0.849	0.787–0.910	0.59	77.3	81.7	0.262

Cut-off, cut off value; AUC, area under the curve; CI, confidence interval; SE, sensitivity; SP, specificity; MMP-7, matrix metalloproteinase-7; APRI, aspartate aminotransferase-to-platelet ratio index; FIB-4, fibrosis-4 index.

### 3.4 Correlation of serum MMP-7 with clinical and fibrosis biomarkers

**Figure 4:**
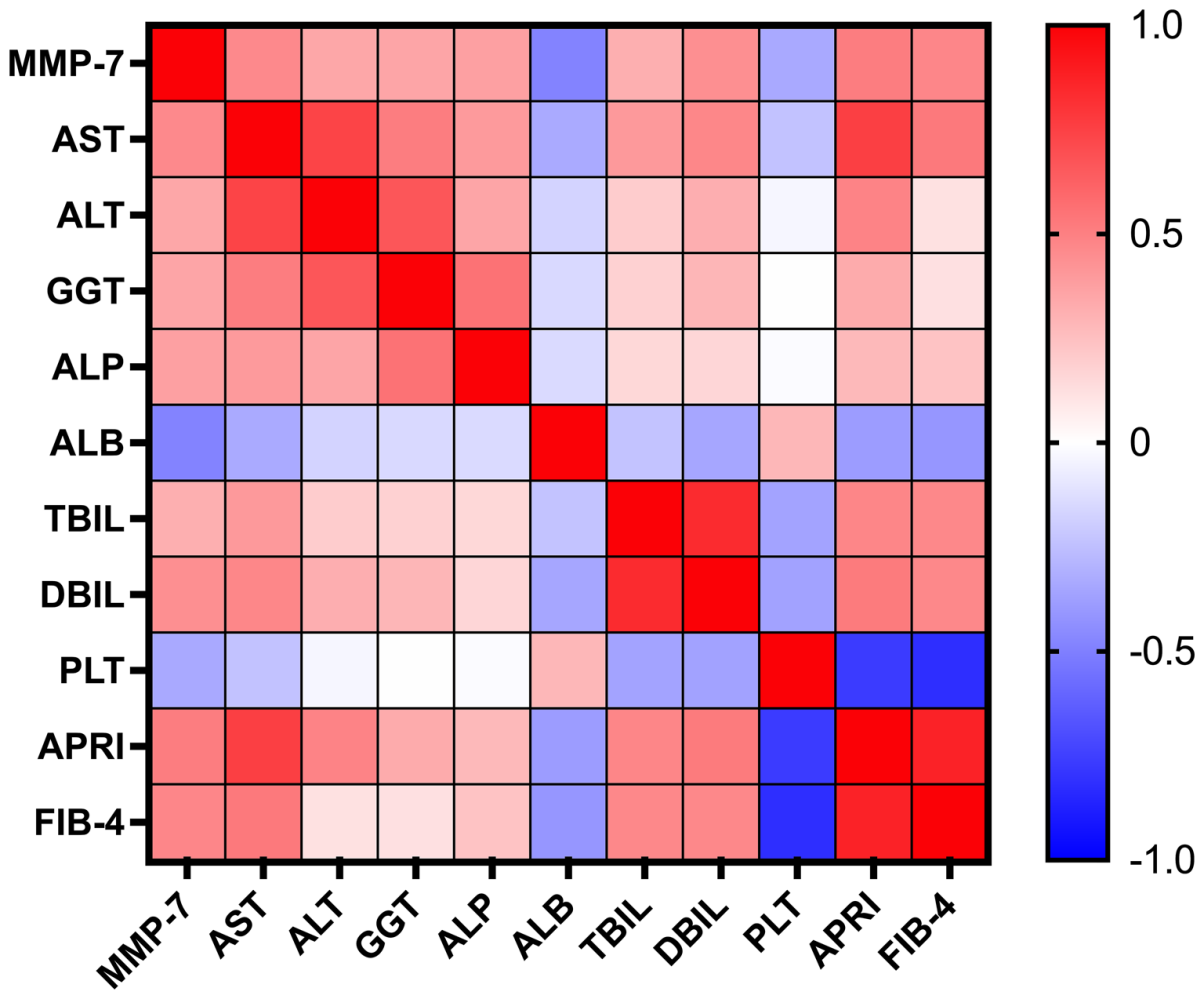
Correlation heatmap between serum MMP-7 and clinical parameters in PBC patients. Color scale indicates correlation strength and direction: red represents positive correlation, blue represents negative correlation, with intensity corresponding to magnitude. AST, aspartate aminotransferase; ALT, alanine aminotransferase; GGT, gamma-glutamyltransferase; ALP, alkaline phosphatase; ALB, albumin; TBIL, total bilirubin; DBIL, direct bilirubin; PLT, platelet; APRI, aspartate aminotransferase-to-platelet ratio index; FIB-4, fibrosis-4 index; MMP-7, matrix metalloproteinase-7.

Serum MMP-7 levels exhibited significant correlations with key clinical parameters in patients with PBC. As visualized in the correlation heatmap (Figure 4), MMP-7 demonstrated strong positive correlations with established non-invasive fibrosis indices, namely APRI and FIB-4. Furthermore, it was positively correlated with markers of hepatocyte injury (e.g., AST and ALT) and cholestasis (e.g., GGT, ALP, TBIL, and DBIL). Conversely, significant inverse correlations were observed with albumin and platelet count. All reported correlations were statistically significant at *p* < 0.01. The complete Spearman’s correlation matrix is provided in Table 3.

**Table 3:** Correlation matrix of MMP-7 with clinical and fibrosis indices in the PBC cohort.

Variable	MMP-7	AST	ALT	GGT	ALP	ALB	TBIL	DBIL	PLT	APRI	FIB-4
MMP-7	–										
AST	0.463**	–									
ALT	0.347**	0.739**	–								
GGT	0.353**	0.510**	0.667**	–							
ALP	0.372**	0.397**	0.355**	0.553**	–						
ALB	−0.489**	−0.336**	−0.175*	−0.150*	−0.146	––					
TBIL	0.315**	0.402**	0.198**	0.180*	0.153*	−0.239**	–				
DBIL	0.440**	0.474**	0.318**	0.288**	0.161*	−0.350**	0.837**	–			
PLT	−0.339**	−0.241**	−0.039	0	−0.016	0.283**	−0.364**	−0.367**	–		
APRI	0.511**	0.755**	0.487**	0.331**	0.276**	−0.390**	0.476**	0.521**	−0.769**	–	
FIB-4	0.475**	0.526**	0.117	0.119	0.235**	−0.415**	0.468**	0.469**	−0.823**	0.870**	–

AST, aspartate aminotransferase; ALT, alanine aminotransferase; GGT, gamma-glutamyltransferase; ALP, alkaline phosphatase; ALB, albumin; TBIL, total bilirubin; DBIL, direct bilirubin; PLT, platelet; APRI, aspartate aminotransferase-to-platelet ratio index; FIB-4, fibrosis-4 index; MMP-7, matrix Metalloproteinas0e-7. **p < 0.01; *p < 0.05. No adjustments were made for multiple comparisons; findings should be considered hypothesis-generating.

### 3.5 Correlation between serum MMP-7 levels and Ludwig stage

**Figure 5:**
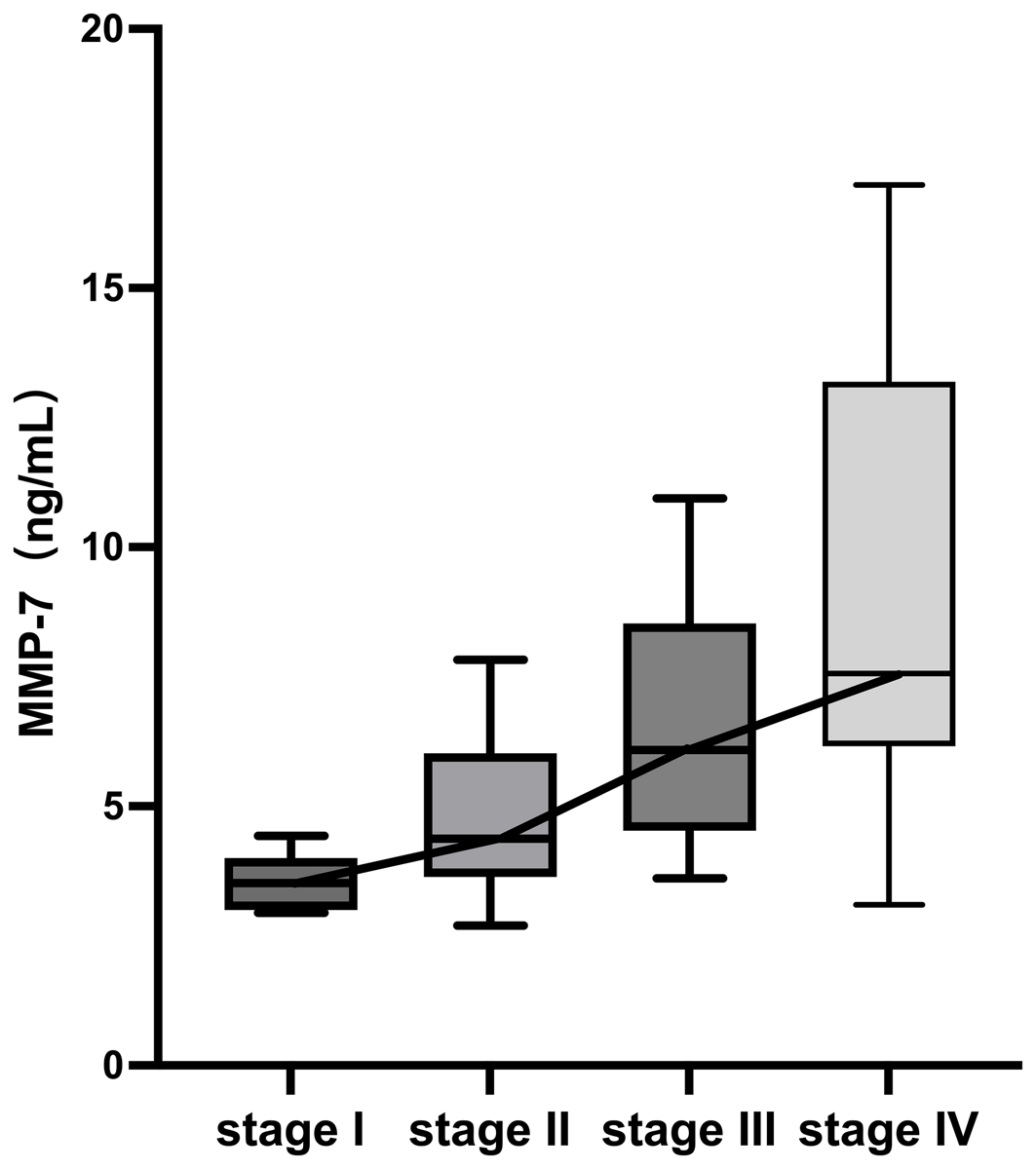
Correlation between serum MMP-7 levels and histological stage in PBC.

The relationship between serum MMP-7 concentration and the degree of liver fibrosis was further measured by analyzing its correlation with the histological Ludwig stage in the biopsy-confirmed subgroup (*n* = 65). Serum MMP-7 levels increased progressively with advancing Ludwig stage and demonstrated a strong positive correlation with the histological stage (*r* = 0.699, *p* < 0.001; Figure 5).

### 3.6 Dynamic changes in serum MMP-7 during UDCA treatment

To evaluate the potential of serum MMP-7 for monitoring treatment response, we analyzed its dynamics in a subgroup of PBC patients (*n* = 33) receiving standard UDCA therapy.

**Figure 6:**
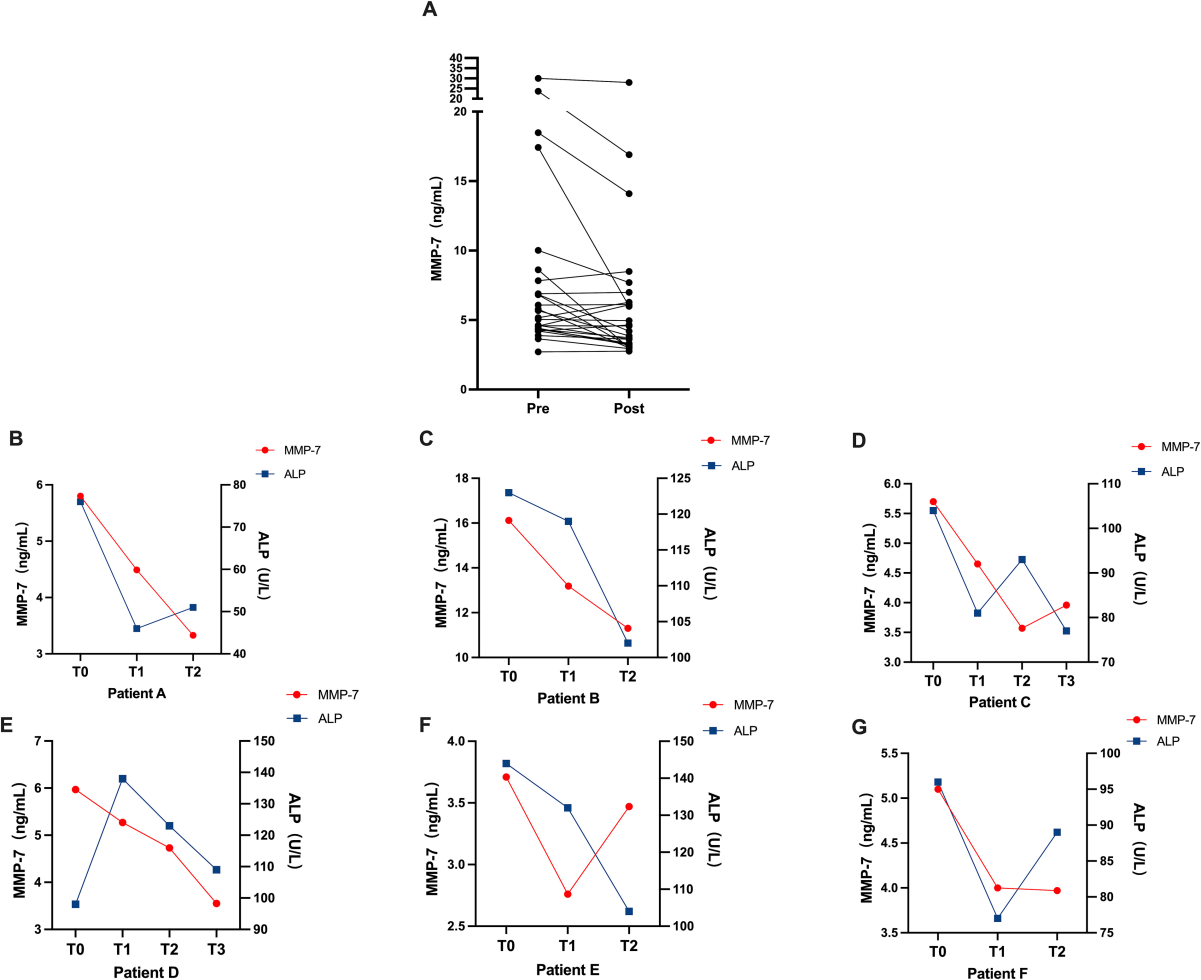
Serum MMP-7 decreases after UDCA treatment and parallels ALP in PBC. **(A)** Paired serum MMP-7 levels in 27 PBC patients before and after 3–4 months of UDCA therapy. **(B–G)** Serial serum MMP-7 (red lines) and ALP (blue lines) in six PBC patients monitored during UDCA treatment. Measurements were obtained at baseline (T0) and at approximately 3–4 month intervals thereafter (T1-T3).

Paired analysis of 27 patients with measurements at baseline and after 3–4 months of UDCA revealed a significant decrease in serum MMP-7 levels (*p* < 0.01; Figure 6A, Table 4). The response was heterogeneous: MMP-7 concentrations decreased in 20 patients but increased in the remaining 7. Consistent with expected biochemical improvement, levels of AST, ALT, GGT, and ALP also decreased significantly from baseline (*p* < 0.05; Table 4).

**Table 4:** Comparison of MMP-7 and other indicator levels in PBC patients before and after treatment.

Indicator	Pre	Post	Statistical value	*p* value
MMP-7 (ng/mL)	5.43 (4.34–8.03)	4.39 (3.25–6.46)	*Z* = −3.048	0.002
AST (U/L)	25 (21–39.25)	24 (19–29)	*Z* = −2.649	0.008
ALT (U/L)	25.5 (14.75–43.75)	22.5 (15.5–31)	*Z* = −1.736	0.083
GGT (U/L)	43.00 (18.75–111.75)	31.5 (17–83.75)	*Z* = −2.073	0.038
ALP (U/L)	112.00 (77.5–163.5)	105.5 (68.75–162.75)	*Z* = -2.409	0.016
ALB (g/L)	43.3 (39.35–45.21)	44.15 (39.90–46.30)	*Z* = -0.889	0.374
TBIL (μmol/L)	14.09 (11.13–26.15)	12.52 (10.57–18.40)	*Z* = −0.698	0.485
DBIL (μmol/L)	5.07 (3.58–8.76)	4.40 (3.13–7.60)	*Z* = −0.431	0.667
PLT (×10^9^/L)	204 (163.75–259.50)	217.00 (166.25–280.75)	*Z* = −1.305	0.192
APRI	0.38 (0.28–0.67)	0.33 (0.22–0.47)	*Z* = −1.740	0.082
FIB-4	1.33 (1.10–2.56)	1.26 (0.79–2.49)	*Z* = −1.435	0.151

AST, aspartate aminotransferase; ALT, alanine aminotransferase; GGT, gamma-glutamyltransferase; ALP, alkaline phosphatase; ALB, albumin; TBIL, total bilirubin; DBIL, direct bilirubin; PLT, platelet; APRI, aspartate aminotransferase-to-platelet ratio index; FIB-4, fibrosis-4 index; MMP-7, matrix metalloproteinase-7. No adjustments were made for multiple comparisons; findings should be considered hypothesis-generating.

In the 6 patients who underwent serial monitoring over time, serum MMP-7 levels demonstrated a dynamic decline that paralleled the reduction in ALP (Figures 6B–G).

## 4 Discussion

PBC is a cholestatic liver disease characterized by immune-mediated destruction of interlobular bile ducts, ultimately leading to fibrosis and cirrhosis (1). While MMP-7 has emerged as a promising biomarker for fibrosis in hepatocyte-centric diseases like NAFLD, its role in PBC—a disease centered on biliary injury—remains poorly defined (22, 23). In this study, we systematically evaluated serum MMP-7 in a Chinese PBC cohort. Our key findings are: (1) Serum MMP-7 levels are significantly elevated in PBC and increase with disease severity; (2) MMP-7 demonstrates excellent diagnostic accuracy for identifying advanced fibrosis and cirrhosis, outperforming conventional scores APRI and FIB-4; (3) Its levels correlate strongly with cholestatic markers and decrease significantly after UDCA treatment, paralleling biochemical response. These results collectively position serum MMP-7 as a novel, pathophysiology-anchored biomarker for stratifying fibrosis and monitoring treatment response in PBC.

The superior performance of MMP-7 in PBC likely stems from its unique origin and dual role in disease pathogenesis, as supported by our findings and the existing literature. Our study confirmed significant elevation of serum MMP-7 levels in PBC patients, with even higher concentrations observed in those with cirrhosis or advanced fibrosis (24). This aligns with previous reports indicating upregulated MMP-7 in cirrhosis (23). Unlike generic fibrosis markers, MMP-7 in the hepatobiliary system is predominantly expressed by cholangiocytes, the primary target in PBC’s chronic non-supportive destructive cholangitis (1). This provides a direct pathophysiological basis for the strong correlation we observed between serum MMP-7 and markers of cholestasis and biliary injury (ALP, GGT, TBIL). Importantly, MMP-7 also correlated significantly with markers of hepatocellular injury (AST, ALT). This broader correlation profile suggests that MMP-7 is not only a direct reporter of the initiating biliary insult but also reflects the subsequent hepatocellular damage characteristic of progressive PBC, positioning it as an integrative biomarker of global hepatic injury. This distinct correlation profile suggests that in PBC, elevated serum MMP-7 primarily reflects direct, real-time damage to the biliary epithelium—the primary disease activity—rather than being merely a secondary consequence of established fibrosis (25, 26).

Consequently, MMP-7 emerges as a critical molecular link connecting the initial biliary insult to the subsequent fibrogenic cascade. Its role extends beyond being a passive biomarker. Evidence of its co-localization in areas of liver injury and fibrosis suggests MMP-7 actively participates in promoting inflammation and fibrogenesis (17). The mechanisms are multifaceted. In addition to degrading basement membrane components, MMP-7 may activate pro-fibrotic pathways (e.g., TGF-*β*1/Smad) in periductal hepatic stellate cells—whose activation further elevates MMP-7 levels—and modulate the inflammatory microenvironment (25, 26). Furthermore, insights from fibrotic pathologies in other organs suggest MMP-7 can promote inflammation and fibrosis by facilitating immune cell migration, promoting angiogenesis (27), mediating apoptosis (28), and participating in Wnt/β-catenin signaling (29). While these mechanisms warrant further validation in hepatic fibrosis, they provide a plausible framework for its pro-fibrotic actions. This direct entanglement within the PBC-relevant pathogenic cascade—from initial cholangiocyte damage to downstream fibrotic signaling—provides a compelling biological rationale for why MMP-7 outperforms conventional fibrosis scores derived from viral hepatitis models.

Indeed, our study demonstrates that a single measurement of serum MMP-7 surpasses the diagnostic utility of APRI and FIB-4 for detecting advanced fibrosis in PBC. However, direct comparison with vibration-controlled transient elastography is lacking in this study, which warrants further investigation. This is not surprising, as APRI and FIB-4 were developed in cohorts with predominant hepatocyte injury (HCV/HBV) and lack sensitivity to the biliary-driven pathophysiology of PBC (30–32). Studies have shown their suboptimal diagnostic performance in autoimmune liver diseases (33). Notably, combining MMP-7 with these scores did not improve diagnostic accuracy, underscoring its potential as a standalone, self-contained biomarker (6, 34, 35). This simplicity is a significant clinical advantage, potentially replacing complex calculations with a single blood test for efficient fibrosis risk stratification. More importantly, the diagnostic performance of MMP-7 (AUC 0.877 for advanced fibrosis) is promising and warrants further comparison with vibration-controlled transient elastography, the current non-invasive gold standard, in future studies (36). The strong correlation we observed between serum MMP-7 and the histological Ludwig stage (*r* = 0.699) further validates its accuracy against the tissue reference, which is consistent with recent findings (24). These data advocate for the inclusion of serum MMP-7 in clinical algorithms to identify patients with advanced disease, potentially sparing those with early-stage PBC from unnecessary liver biopsy.

Beyond cross-sectional diagnosis, our longitudinal data reveal the dynamic potential of MMP-7 for monitoring therapeutic response. In UDCA-treated patients, a significant decline in serum MMP-7 levels paralleled the improvement in classic cholestatic and injury biomarkers (AST, ALT, GGT, ALP) (37). The rapid decrease in MMP-7 within 3–4 months of UDCA therapy can be explained by its cellular origin and pathophysiological role. MMP-7 is primarily produced by injured cholangiocytes, serving as a direct molecular readout of biliary epithelial damage. UDCA alleviates cholangiocyte injury by improving bile flow and reducing hydrophobic bile acid toxicity (1, 4, 38). As biliary inflammation subsides, transcriptional activation of MMP-7 diminishes, leading to a rapid decline in serum levels. This early response suggests that MMP-7 may serve as a sensitive indicator of treatment-related amelioration of biliary injury, potentially preceding conventional biochemical markers (39). Given that persistent biliary injury drives fibrosis (40), the early decline in MMP-7 may reflect mitigation of fibrogenic signals, underscoring its potential as a surrogate marker for treatment efficacy at the tissue level. Notably, we observed heterogeneous trajectories, with MMP-7 levels increasing in a subset of patients. This heterogeneity mirrors the well-established variability in UDCA biochemical response. Early identification of patients with rising or non-declining MMP-7 levels could, therefore, provide a valuable signal for inadequate response to UDCA monotherapy, informing timely clinical decisions regarding escalation to second-line therapies before fibrosis progresses (41, 42).

Our study has several limitations. First, its single-center, retrospective design and exclusive Chinese cohort necessitate validation in multi-ethnic, prospective populations. Second, while we employed rigorous clinical criteria, the partial reliance on imaging for cirrhosis diagnosis may introduce spectrum bias. Third, the relatively short treatment follow-up period precludes direct correlation between MMP-7 dynamics and long-term hard endpoints like decompensation or survival. Fourth, the absence of a disease control group—such as patients with primary sclerosing cholangitis, biliary obstruction, or drug-induced cholestasis—limits our ability to determine whether elevated MMP-7 is specific to PBC or reflects a general response to biliary injury. Fifth, vibration-controlled transient elastography was not systematically performed in this cohort, precluding direct comparison between MMP-7 and this non-invasive gold standard. Sixth, as this was an exploratory study intended for hypothesis generation rather than confirmatory validation, no post-hoc pairwise subgroup comparisons were conducted, and no adjustments for multiple testing were applied for correlation and paired comparisons, which may increase the risk of type I error. The findings should thus be interpreted with caution. Seventh, the MMP-7 assay kit used in this study is intended for research use only and has not yet been approved for clinical diagnostic use. Nevertheless, the promising performance observed in this study supports the need for further validation and eventual clinical assay development. Eighth, serum bile acid levels and pruritus scores were not systematically recorded in this retrospective cohort, limiting our ability to explore correlations between MMP-7 and these clinically relevant parameters. Future prospective studies should incorporate these assessments to further validate the clinical utility of MMP-7.

Future research should: (1) Conduct large-scale, prospective studies to confirm the diagnostic and prognostic utility of MMP-7; (2) Explore the synergy of combining serum MMP-7 with vibration-controlled transient elastography for enhanced non-invasive staging; (3) Investigate the long-term predictive value of serial MMP-7 measurements for fibrosis regression, disease progression, and clinical outcomes; (4) Include comparator cohorts with other cholestatic liver diseases to establish the specificity of MMP-7 for PBC.

## 5 Conclusion

In conclusion, this exploratory study provides preliminary evidence that serum MMP-7—a biomarker originating from injured cholangiocytes—may serve as a pathophysiology-relevant biomarker in PBC, directly reflecting biliary injury and fibrogenesis. This study systematically evaluates MMP-7 in PBC, demonstrating promising diagnostic performance for advanced liver fibrosis that may complement existing non-invasive tools such as APRI, FIB-4, and elastography. Its dynamic changes following UDCA therapy further suggest potential utility in monitoring treatment response. These findings warrant validation in future prospective studies, and if confirmed, integration of serum MMP-7 into clinical management algorithms could enhance risk stratification and therapeutic decision-making in PBC.

## Data Availability

The original contributions presented in the study are included in the article/supplementary material, further inquiries can be directed to the corresponding author.
